# Anti‐inflammatory effect from extracts of Red Chinese cabbage and Aronia in LPS‐stimulated RAW 264.7 cells

**DOI:** 10.1002/fsn3.1472

**Published:** 2020-02-24

**Authors:** Jung Hyun Kwak, Yoonji Kim, Soo In Ryu, Minho Lee, Hyo‐Jeong Lee, Yong Pyo Lim, Jean Kyung Paik

**Affiliations:** ^1^ Department of Food and Nutrition Eulji University Seongnam Korea; ^2^ Nutrition Team Wonkwang University Sanbon Hospital Gunpo Korea; ^3^ Department of Food Technology and Services Eulji University Seongnam Korea; ^4^ Department of Science in Korean Medicine Graduate School Kyung Hee University Seoul Korea; ^5^ Molecular Genetics and Genomics Laboratory Department of Horticulture Chungnam National University Daejeon Korea

**Keywords:** anthocyanins, anti‐inflammatory effect, Aronia, Red Chinese cabbage

## Abstract

A chronic inflammatory environment facilitates tumor growth and proliferation. Fruits and vegetables are important sources of anthocyanins, polyphenols, and other biologically active substances that can favorably affect the pathogenesis of cancer. The objective of the study was to investigate the anti‐inflammatory effects of Red Chinese cabbage (RC) and mixture of commercial Red Chinese cabbage leaves and Aronia fruits (ARC) in LPS‐stimulated RAW 264.7 cells. The RAW 264.7 cells were cultured and measured the cytotoxicity by using an MTT assay. The inflammatory markers, such as nitrite, IL‐6, and TNF‐alpha expression, were evaluated using ELISA, and protein expression of inflammatory markers like iNOS and COX‐2 was analyzed using Western blot. MTT assays showed that pretreatment of RAW 264.7 cells with RC and ARC did not change cell growth or cytotoxicity. We also found that ARC extracts reduced inflammation‐related biomarker (TNF‐a, IL‐6, and NO) production and gene expression (iNOS, COX‐2). Our results suggested that ARC has good anti‐inflammatory properties compared with RC that maybe used as potential nutrients for treating inflammatory diseases.

## Graphical abstract

The extracts of Red Chinese cabbage and Aronia have good anti‐inflammatory properties compared with Red Chinese cabbage that maybe used as potential nutrients for treating inflammatory diseases.

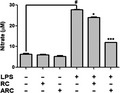

## 1 INTRODUCTION

Cancer is currently one of the foremost public health concerns in Korea, and the death rate from cancer has increased steadily over the past decade (Jung et al., 2017).

Modern lifestyles such as excessive alcohol consumption and too much dietary fat intake can increase the risk of cancer (Park, Lee, & Kim, 2018; Vieira, Tobar, Dardes, Claudio, & Thuler, 2018), whereas fruits and vegetables are important sources of fiber, vitamins, anthocyanins, and other biologically active substances that can favorably affect the pathogenesis of cancer (Farvid et al., 2016; Li et al., 2016).

Chinese cabbage (*Brassica rapa L.* ssp. *pekinensis)* is a leafy vegetable crop belonging to the genus *Brassica* and family *Brassicaceae*, which is widely consumed in Asian countries, particularly in Korea (Rubab et al., 2018). Recently, Red Chinese cabbages (RC) (*Brassica rapa* L) are produced by crossing red cabbage (*Brassica oleracea* L. var. *captita f. rubra*) with Chinese cabbage (*Brassica rapa* L. ssp. *pekinensis*) (Jiang et al., 2013). It is rich in anthocyanins and characterized by red color on the outside and inside.

Aronia fruits, with the common name chokeberry, belong to the Aronia genus of the *Rosaceae* family, *Maloideae* subfamily. It is a rich source of polyphenolic compounds, which are one of the most important and vital natural antioxidants (Dei Cas & Ghidoni, 2018; Jurikova et al., 2017).

A review study reported that the synergistic effects of phytochemical extracts from fruits and vegetables have strong antioxidant and antiproliferative activities, and the major part of total antioxidant activity is from the combination of phytochemicals (Liu 2004). Studies for each single food such as Red Chinese cabbage, or Aronia related to anti‐inflammation and antioxidation are reported (Joo et al., 2018; Kokotkiewicz, Jaremicz, & Luczkiewicz, 2010), but the anti‐inflammatory effects with the mixture of Red Chinese cabbage and Aronia fruits have not been studied. Therefore, our objective was to investigate the anti‐inflammatory effect of mixtures of Red Chinese cabbage and Aronia (ARC) in LPS‐stimulated RAW 264.7 cells.

## 2 MATERIALS AND METHODS

### 2.1 Sample preparation

We obtained Aronia (*Aronia melanocarpa* (Michx.) Elliot) frozen fruits from a plantation farm located in Yongin (Gyeonggi‐do, Korea) and ground it in a grinder (FM‐909W, Hanil Co., Sejong, Korea). And RC was purchased from a plantation farm located in Bugil‐myeon (Haenam‐gun, Jeollanam‐do, Korea), cultivated using the seeds of commercial cultivar (Kwonnong 3 Ho), distributed by Kwonnong Seed Company (Chungju, Korea), and removed the foreign matter, and cut the whole RC sample into one centimeter pieces.

### 2.2 Preparation of extracts

RC extraction was diluted with 95% fermented ethanol at a sample:alcohol ratio (1:10) and extracted the filtrate for 24 hr. We prepared the ARC mixture (Red Chinese cabbage and Aronia fruit extracts at a ratio of 2:8) further, diluted with 95% fermented ethanol at a sample:alcohol ratio (1:10), and extracted the filtrate for 24 hr. To remove impurities, the solvent extract was filtered through a cotton fabric (No. 1, Whatman International. Ltd., Leicestershire, England). The extract was allowed to evaporate at room temperature using a rotor evaporator (EYELA/N1000, Tokyo Rikakikai Co.) under reduced pressure to obtain dry residue, and further, it is stored in a cool and dark condition. Finally, the dry residue is dissolved in dimethyl sulfoxide (DMSO) and stored at −20°C.

### 2.3 Cell culture

The RAW 264.7 murine cell line of macrophage (Korean cell‐line bank) was cultured in Dulbecco`s modified Eagle`s medium (DMEM, Gibco) supplemented with 10% inactivated fetal bovine serum and 1% penicillin–streptomycin. And the cell culture is maintained at humidified atmosphere with 5% CO_2_ at 37°C.

### 2.4 Cytotoxic assay

The cytotoxicity was measured by using an 3‐(4,5‐dimethylthiazol‐2‐yl)‐2,5‐diphenyl‐2H‐tetrazolium bromide (MTT) assay and aliquot cells (RAW 264.7 cells) at a density of 10^5^ cells/ml into 96‐well plates and incubated them for 20 hr. After incubation, the plates were treated with different concentrations of RC and ARC extracts and incubated for 22 hr with 5% CO_2_ at 37°C. Each well was added with 5 mg/ml thiazolyl blue tetrazolium bromide (MTT, Sigma‐Aldrich Co.). After incubation for 2 hr in CO_2_ incubator, the supernatant was removed after centrifugation at 4℃ at 1747 *g* for 10 min. Each well then had dimethyl sulfoxide added (Sigma‐Aldrich Co.), and the plates were identified by a microplate reader (Model 550, Bio‐Rad) at 540 nm.

### 2.5 Nitrite measurement

The generation of nitric oxide (NO) was measured by Griess reaction. The RAW 264.7 cells (1 × 10^6^ cells/ml) were aliquot in six‐well plate and incubated them for 20 hr. Followed by pretreatment of the cells with different concentrations of RC and ARC, 1 μg/ml of lipopolysaccharide (LPS) was added to 250 μg/ml of each sample. After 24 hr incubation, 100 μl culture was mixed with an equal volume of Griess reagent at room temperature for 10 min. The absorbance of the mixture was measured by a microplate reader at 540 nm. We performed all of the experiments in triplicate.

### 2.6 Cytokine expression by ELISA

Enzyme‐linked immunosorbent assay (ELISA) was used to evaluate tumor necrosis factor‐alpha (TNF‐α) and interleukin‐6 (IL‐6) expression. The RAW 264.7 cells (1 × 10^6 ^cells/ml) were aliquot into six‐well plate and incubated for overnight; 1 μg/ml lipopolysaccharide (LPS) was added to 250 μg/ml of each sample. After incubation for 24 hr, the concentration of TNF‐α and IL‐6 in the cell‐free supernatant (i.e., CM) was assessed by using the ELISA kit (Cloud‐Clone Crop) according to the manufacturer's instructions.

### 2.7 Western blot analysis

Initially, the cell pellets were washed in ice‐cold PBS buffer, followed by lysis using Radioimmunoprecipitation assay (RIPA) buffer (Pierce Biotechnology) and then measured protein concentration by DC^TM^ assay (Bio‐Rad). A protein sample with 30 μg was subjected to electrophoresis using 10% SDS‐PAGE in running buffer at 120 V for 2 hr and electroblotting onto a PVDF (polyvinylidene difluoride) membrane. The membrane was then blocked in the solution that contains 5% nonfat milk, 0.1% (v/v) Tris‐buffered saline, and Tween‐20 (TBS‐T) for 1 hr at room temperature. After blocking, we then washed the membranes thrice with TBS‐T for 10 min. Then, each membrane was added with primary antibodies (1:1,000; diluted with 1% nonfat milk in TBS‐T) specific with inducible nitric oxide synthase (iNOS) (AVIVA) and cyclooxygenase‐2 (COX‐2) (Santacruz) and incubated overnight at 4°C on shaker. β‐actin was used as the internal control. After incubation, the membrane was washed with TBS‐T thrice (10 min/time) and again incubated by adding secondary antibodies (anti‐rabbit and anti‐mouse IgG horseradish peroxidase) at a ratio of 1:5,000 (3% nonfat milk: TBS‐T) for 2 hr. Followed by washing the membrane in TBS‐T thrice, the last washing was done using TBS for 10 min. Finally, the protein expression was detected by an enhanced chemiluminescence (ECL) immunoassay and Western blotting detection reagents (Amersham, GE Healthcare).

### 2.8 Statistical analysis

Data analyses were done using SPSS software version 17.0 and represented data as means ± standard deviation (*SD*). An independent *t* test was used to compare means, and *p* < .05 was considered to indicate statistically significant difference.

## 3 RESULTS

### 3.1 Cytotoxic assay

**Figure 1:**
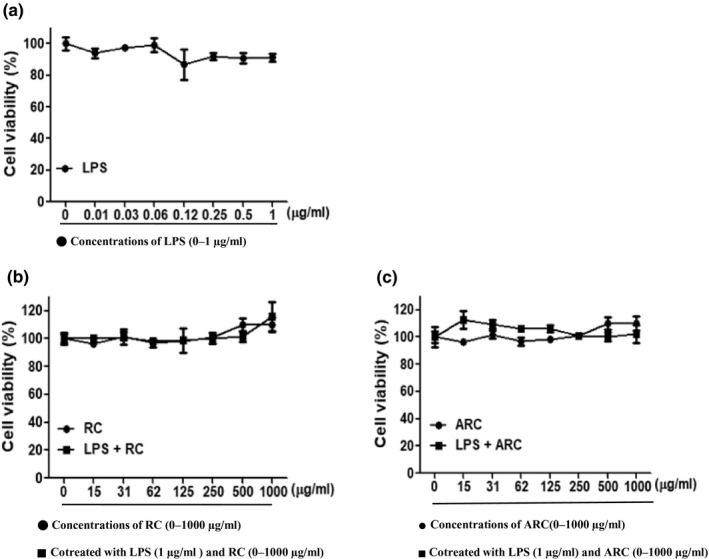
Effect of RC and ARC on cells viability in RAW 264.7 cells: The effect of RC and ARC at various concentrations (0–1,000 μg/ml) on cell viability in RAW 264.7 was measured using an MTT assay. (a) Treated with LPS at various concentrations (0–1 μg/ml) for 24 hr. (b) Treated with RC at various concentrations (0–1,000 μg/ml) for 24 hr. Cotreated with LPS (1 μg/ml) and RC (0–1,000 μg/ml) for 24 hr. (c) Treated with ARC at various concentrations (0–1,000 μg/ml) for 24 hr. Cotreated with LPS (1 μg/ml) and ARC (0–1,000 μg/ml) for 24 hr

Figure 1 shows the effect of RC and ARC at various concentrations (0, 15, 31, 62, 125, 250, 500, and 1,000 μg/ml) on cell viability of RAW 264.7 cells. The nonspecific cell toxicity of RC and ARC was measured using the MTT assay. From the results of cell viability assay, it was evident that even at high concentration, RC and ARC did not show cytotoxicity on RAW 264.7 cells. When LPS was treated with RC and ARC, the results were similar to those of RC and ARC alone. In addition, LPS treatment alone showed 90% cell viability at high concentrations.

### 3.2 Effect of RC and ARC on NO production

**Figure 2:**
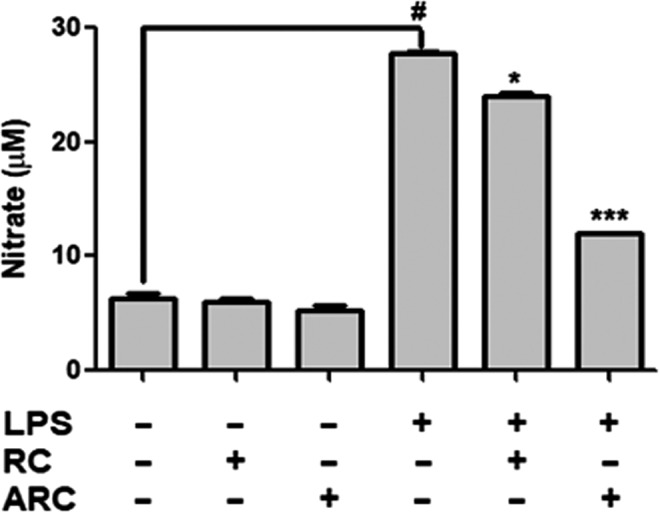
Effect of RC and ARC on LPS‐stimulated NO production in RAW 264.7 cells: RAW 264.7 cells were treated with LPS (1 μg/ml), RC (250 μg/ml), and ARC (250 μg/ml) concentrations for 24 hr. The data are expressed as mean ± *SD* (*n* = 3). # *p < *.05, statistically different from control (nontreat) using *t* test. *p < .05, ***p < .001, statistically different from LPS control using *t* test

Figure 2 presents the effect of RC and ARC on NO production in RAW 264.7 cells. Cells treated with LPS increased NO production gradually, and the production reduced with treatment of RC and ARC extracts. The degree of NO production was remarkably decreased in ARC‐treated cells more than by RC‐treated cells.

### 3.3 Effect of RC and ARC on the inflammatory cytokines

**Figure 3:**
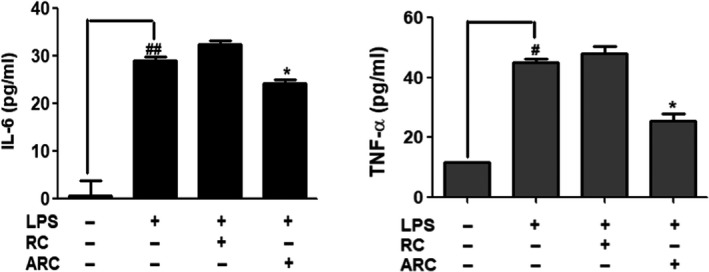
Effect of RC and ARC on LPS‐induced cytokines of IL‐6 and TNF‐α in RAW 264.7 cells, which were treated with LPS (1 μg/ml), RC (250 μg/ml), and ARC (250 μg/ml) concentrations for 24 hr. The data are expressed as mean ± *SD* (*n* = 3). # *p < *.05, ## *p < *.01, statistically different from control (nontreat) using *t* test. *p < .05, statistically different from LPS control using *t* test

Figure 3 presents the inhibitory effect of IL‐6 and TNF‐α on the inflammatory cytokines of RC and ARC extracts. Treatment with LPS induced an increase in the concentrations of inflammatory cytokines (IL‐6 and TNF‐α), and the production reduced with the treatment of ARC. When treated with ARC and LPS, IL‐6 and TNF‐α concentrations were 24 and 25 pg/ml, respectively, lower than when treated with LPS alone (28 and 44 pg/ml). However, there was no significant effect when treated with RC.

### 3.4 Effect of RC and ARC on gene expression

**Figure 4:**
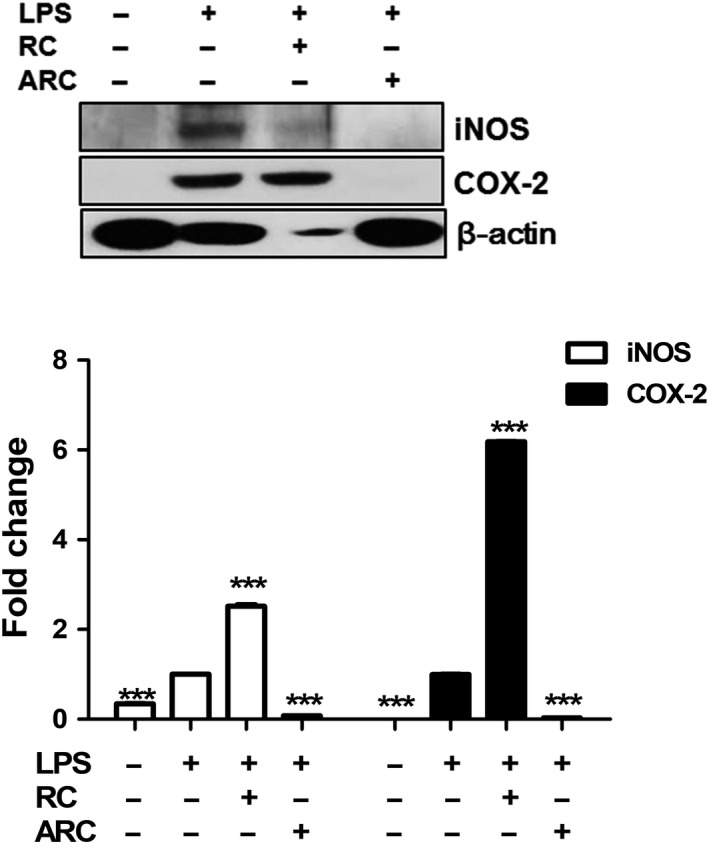
Effect of RC and ARC on LPS‐induced COX‐2 and iNOS expression in RAW 264.7 cells, which were treated with LPS (1 μg/ml), RC (250 μg/ml), and ARC (250 μg/ml) concentrations for 24 hr. The data are expressed as mean ± *SD* (*n* = 3). ***p < .001, statistically different from LPS control using *t* test

Figure 4 shows the effect of RC and ARC on LPS‐induced COX‐2 and iNOS expression using a Western blot analysis. Incubation of RAW 264.7 cells with LPS for 24 hr stimulated the expression of iNOS and COX‐2 protein, by addition of ARC suppressed the expression of iNOS and COX‐2 protein. However, when treated with RC and LPS, the expression of iNOS and COX‐2 increased.

## 4 DISCUSSION

The anti‐inflammatory efficacy of RC and ARC in LPS‐stimulated RAW 264.7 cells was evaluated. The inflammatory markers, such as NO, IL‐6, and TNF‐α expression, were evaluated using ELISA, and protein expression of inflammatory markers like iNOS and COX‐2 was analyzed using Western blot. In conclusion, our findings indicate that ARC could attenuate the inflammatory response by suppressing the iNOS and COX‐2 genes and mediators in RAW 264.7 cells.

According to a review study, Lee et al. reported on the effects of anthocyanins‐rich food on attenuating inflammation in cells, animals, and humans (Lee et al., 2017). Particularly, anthocyanin mixtures such as red cabbage, microgreens, blueberry, blackcurrant, cherry, and chokeberry had higher clinical efficacy than single anthocyanins. In our study, the results were similar to those reported in previous reviews.

In previous studies, Joo et al. evaluated the effects of anthocyanin‐rich extract from Red Chinese cabbage (RC) on vascular inflammation in endothelial cells and apolipoprotein E‐deficient mice. They reported that the major anthocyanin of the extract is cyanidin [186 mg/g (dry weight)] and found that the RC‐mediated suppression of transcription and expression of adhesion molecules correlated with lowering the risk of vascular inflammatory disease (Joo et al., 2018). Lee et al. (2018 investigated and compared the contents of phenolic compounds in RC and typical green Chinese cabbage (GC). They reported that the RC contained more phenolic compounds than the traditional GC, and cyanidin is found to be a major anthocyanin pigment found in RC. Also, Ha et al. reported that the extract of RC contained bioactive compounds such as polyphenol and flavonoids, which are effective as antioxidants and anti‐inflammation in an in vitro study (Ha & Lee 2014).

In the preceding studies, there was a positive report on the antioxidant and anti‐inflammatory effects of RC. However, when RC alone in our study, there was no significant change in inflammatory markers such as IL‐6 and TNF‐a except that NO was decreased and iNOS and COX‐2 gene expression increased. These results verify the effects of RC at 250 μg/ml concentrations. The effects at other concentrations should be confirmed by further study.

*Aronia Melanocarpa*, with the common name black chokeberry, is a shrub of the Rosaceae family (Kokotkiewicz et al., 2010). Aronia fruits have a high content of bioactive components, such as polyphenol (the range of 690–2,560 mg gallic‐acid equivalents in 100 g fresh weight) than most other berries (Jakobek, Šeruga, Medvidović‐Kosanović, & Novak, 2007; Rop et al., 2010). Also, Aronia includes anthocyanins, procyanidins, and phenolic acids (Kulling & Rawel, 2008; Valcheva‐Kuzmanova & Belcheva, 2006). The antioxidant and anti‐inflammatory properties of Aronia fruits are related to prevent the development of chronic diseases such as cardiovascular diseases (Sikora, Broncel, & Mikiciuk‐Olasik, 2014), diabetes (Adisakwattana, Yibchok‐Anun, Charoenlertkul, & Wongsasiripat, 2011), and cancer (Gasiorowski et al., 1997).

The synergistic effects of phytochemical extracts from fruits and vegetables have strong antioxidant and antiproliferative activities, and the major part of total antioxidant activity is from the combination of phytochemicals (Liu 2004). This balanced natural combination of phytochemicals present in fruit and vegetable cannot be completely reproduced in the form of dietary supplements. In a review study, Chiou et al. reported that a combination of phytochemicals can have anti‐inflammatory and anticancer effects by synergy though balancing cytokines‐regulated tumor proproliferative and inflammatory signaling (Chiou, Li, Ho, & Pan, 2018). Therefore, synergistic research of naturally derived fruit and vegetable mixtures is very important and necessary.

Although studies on single foods of Red cabbages or Aronia have been reported, the effects of the complex have not been studied. We verified the effect at the cellular level.

Chronic inflammation may promote the development of tumor growth through a variety of mechanisms, such as stimulating angiogenesis, preventing apoptosis, and promoting proliferation and migration (Allen & Jones, 2015). Inflammatory response regulated by inflammatory mediators (e.g., NO) and proinflammatory cytokines (e.g., TNF‐α, IL‐6). Under inflammatory conditions, iNOS and COX‐2 protein expression are elevated, which facilitates the generation of NO and Prostaglandin F2 alpha (PGF_2α_). iNOS is a target of inflammation‐associated tissue damage (Southan & Szabo, 1996), and COX‐2 is a mediator of inflammation, angiogenesis, and cancer progression (Wang et al., 2017). Also, cytokines that include TNF‐α and IL‐6 are related to the inflammation process and known to typical proinflammatory cytokine with tumor growth effect (Landskron, De la Fuente, Thuwajit, Thuwajit, & Hermoso, 2014).

The findings in our study evidence that ARC extracts significantly inhibit the production of NO, TNF‐α, and IL‐6. Additionally, these were associated with decreased expression of iNOS and COX‐2 protein. To the best of our knowledge, this study is the first to examine the anti‐inflammatory effects of mixtures of ARC on LPS‐stimulated RAW 264.7 cells.

However, in our study, the anti‐inflammatory effect of RC and ARC was verified, but the result of treatment with Aronia extract alone could not be analyzed. Although there are results on the anti‐inflammatory effect of aronia itself (Tunde Jurikova et al., 2017), a comparison of the results of treatment with aronia extract alone will be necessary to validate the results. In addition, accurate content analysis of polyphenols and anthocyanins in single and mixed extracts should be preceded in further study.

In conclusion, our findings indicate that ARC could attenuate the inflammatory response by suppressing the iNOS and COX‐2 genes and mediators in RAW 264.7 cells. However, further studies should be performed to confirm the effect at different concentrations and to verify the effect on the single substance as well as the mixture.

## CONFLICT OF INTEREST

The authors declare no potential conflicts of interest.

## AUTHOR CONTRIBUTIONS

The authors’ responsibilities were as follows: JHK and YK designed and created the study concept; HJL acquired the data and performed the statistical analysis; JHK wrote the paper; SIR, ML, HJL, and YPL contributed critical advice and revisions of the manuscript; JKP had responsibility for the entire contents of the manuscript and obtained funding; JKP supervised the study; and all authors had full access to the study data and read and approved the final manuscript.

## ETHICAL APPROVAL

This study does not involve any human or animal testing.
